# Bioaccesibility, Metabolism, and Excretion of Lipids Composing Spent Coffee Grounds

**DOI:** 10.3390/nu11061411

**Published:** 2019-06-23

**Authors:** Amaia Iriondo-DeHond, Fresia Santillan Cornejo, Beatriz Fernandez-Gomez, Gema Vera, Eduardo Guisantes-Batan, Sergio Gomez Alonso, Manuel Ignacio San Andres, Sebastian Sanchez-Fortun, Laura Lopez-Gomez, Jose Antonio Uranga, Raquel Abalo, Maria Dolores del Castillo

**Affiliations:** 1 Instituto de Investigación en Ciencias de la Alimentación (CIAL) (CSIC-UAM), Campus de la Universidad Autónoma de Madrid, 28049 Madrid, Spain; amaia.iriondo@csic.es (A.I.-D.); fresia.santillan@gmail.com (F.S.C.); b.fernandez@csic.es (B.F.-G.); 2 Departamento de Ciencias Básicas de la Salud, Facultad de Ciencias de la Salud, Universidad Rey Juan Carlos (URJC), Alcorcón, 28922 Madrid, Spain; gema.vera@urjc.es (G.V.); laura.lopez.gomez@urjc.es (L.L.-G.); jose.uranga@urjc.es (J.A.U.); raquel.abalo@urjc.es (R.A.); 3 Instituto Regional de Investigación Científica Aplicada (IRICA), Universidad de Castilla-La Mancha, 13005 Ciudad Real, Spain; eduardo.guisantes@uclm.es (E.G.-B.); sergio.gomez@uclm.es (S.G.A.); 4 Facultad de Veterinaria, Universidad Complutense de Madrid, 28040 Madrid, Spain; misanand@vet.ucm.es (M.I.S.A.); fortun@vet.ucm.es (S.S.-F.)

**Keywords:** bioaccesibility, cafestol, fatty acids, kahweol, lipid excretion, lipid liver biomarkers, lipid metabolism, spent coffee grounds

## Abstract

The bioaccessibility, metabolism, and excretion of lipids composing spent coffee grounds (SCGs) were investigated. An analysis of mycotoxins and an acute toxicity study in rats were performed for safety evaluation. Total fat, fatty acids, and diterpenes (cafestol and kahweol) were determined in SCGs and their digests obtained in vitro. A pilot repeated intake study was carried out in Wistar rats using a dose of 1 g SCGs/kg b.w. for 28 days. Fat metabolism was evaluated by analysis of total fat, cholesterol, and histology in liver. The dietary fiber effect of SCGs was measured radiographically. The absence of mycotoxins and toxicity was reported in SCGs. A total of 77% of unsaturated fatty acids and low amounts of kahweol (7.09 µg/g) and cafestol (414.39 µg/g) were bioaccessible after in vitro digestion. A significantly lower (*p* < 0.1) accumulation of lipids in the liver and a higher excretion of these in feces was found in rats treated with SCGs for 28 days. No lipid droplets or liver damage were observed by histology. SCGs acutely accelerated intestinal motility in rats. SCGs might be considered a sustainable, safe, and healthy food ingredient with potential for preventing hepatic steatosis due to their effect as dietary fiber with a high fat-holding capacity.

## 1. Introduction

Coffee is one of the major plantation crops grown worldwide and one of the most popular beverages consumed all over the world. When coffee is extracted in water for the preparation of the beverage, most of the hydrophobic compounds, such as oils, lipids, triglycerides, and fatty acids as well as insoluble carbohydrates like cellulose and other indigestible sugars remain in the grounds [1].

Spent coffee grounds (SCGs) are the most abundant coffee byproduct (45%) generated during the treatment of coffee powder with hot water to prepare coffee infusion or steam for the instant coffee preparation [2]. The main ingredient present in SCGs is dietary fiber and it can be categorized as antioxidant dietary fiber, useful as a potential dietary supplement [3,4,5]. SCGs dietary fiber is mainly composed of insoluble fiber (88% total dietary fiber), representing 41.6% of the total sample. SCGs are rich in mannose, galactose, glucose, and arabinose polymerized into hemicellulose and cellulose (45.3–51.5%, *w*/*w*) [6,7], and there is also high content of galactomannans [8]. In addition, lignin is also present in a significant amount in SCGs (19–26%, *w*/*w*) [6,9].

The second most predominant ingredient in SCGs are lipids, which range from 10 to 30% [3,4]. The composition of lipids from SCGs slightly differs from that of raw coffee beans, but generally, coffee oil contains mainly triacylglycerols (75%) and unsaponificables, including about 19% total free and esterified diterpene alcohols, about 5% total free and esterified sterols, and very low amounts of other substances such as tocopherols [10]. Diterpenes, cafestol and kahweol, present in the lipid fraction of coffee, are related to increased blood cholesterol, especially in the case of cafestol [11,12]. However, the diterpenes kahweol and cafestol are also known for their potential beneficial physiological effects such as ultraviolet B (UVB) skin protection, anticarcinogenic, anti-inflammatory, and antioxidant activities [13].

The application of SCGs represents a value-added opportunity for coffee byproduct utilization at a very low cost. Several applications have been proposed for SCGs, which include their use as biofuels, biosorbents, animal feed, ceramic manufacturing, and composite materials, among others [14,15]. Nevertheless, these approaches may actually be an inefficient way to use this biomass waste. Considering the chemical composition of SCGs and their related potential health-promoting properties, it might be interesting to maintain the use of SCGs within the food industry sector. Therefore, SCGs have been proposed for use as dietary fiber in bakery products such as bread, cookies, and breakfast cereals, among others, giving rise to healthy and sustainable novel products [4,16]. The second main component of this coffee byproduct is fat, and its bioaccesibility and in vivo effect might be modulated by the dietary fiber composing SCGs. Consequently, the aim of the present study was to determine the in vitro bioaccessibility of lipids and diterpenes (cafestol and kahweol), their impact on liver lipid biomarkers, and their excretion in feces, for the validation of SCGs as a safe, healthy, and sustainable food ingredient with a dietary fiber effect and possessing a high fat-holding capacity.

## 2. Materials and Methods

### 2.1. SCGs Samples

Raw SCGs Robusta species from industrial soluble coffee production were provided by Prosol S.A (Palencia, Spain). SCGs were thermally stabilized by drying in an oven (Memmert, UM 500, Büchenbach, Germany) at 40 °C for 9 h until constant weight was achieved.

### 2.2. Safety of SCGs

#### 2.2.1. Mycotoxins

Aflatoxin B1, enniantin B, and ochratoxin A (OTA) were analyzed by high-performance liquid chromatography–quadrupole-time of flight mass spectrometry (HPLC-QToF-MS) formed by a HPLC 1260 Infinity system (Agilent, Waldbronn, Germany) coupled to a 6545 QToF mass spectrometry detector (Agilent, Singapore)following the procedure described by Iriondo-DeHond et al. (2019) [17].

#### 2.2.2. Acute Toxicity Study

The acute toxicity study was performed following the directions of the OECD (Organization for Economic Co-operation and Development) Test Guidelines 425 (Up and Down Procedure). Healthy 8-week-old nulliparous and nonpregnant female Wistar rats (*Rattus norvegicus albinus*), weighing 180.52 ± 6.42 g at the start of the experiment, were purchased from Charles River (Sant Cugat del Vallés, Spain). This study was approved by the Institutional Animal Ethics Committee (Reg. No. PROEX 011/17) of Community of Madrid, Spain. The animals were kept in a room with a controlled temperature of 22 ± 1 °C in 12 h light–dark cycles and were housed in cages with free access to standard food (A04 Safe Diets, Augy, France) and water ad libitum. A limit test of SCGs dissolved in corn oil was performed at 2000 mg/kg b.w. by gavage as a single dose to one rat. As a control, one rat was dosed with corn oil. For the first 30 min to 4 h, rats were closely observed. If the treated rat survived, 4 additional rats per group were administered SCGs or corn oil under the same conditions. Twenty-four hours after SCGs or corn oil administration and every day for 14 days, body weight changes, signs of toxicity, behavior, and mortality were observed. On the last day of the study, rats were sacrificed in a gas chamber by exposure to excess carbon dioxide (CO_2_). Histopathological examination was carried out in collected organs (heart, lungs, liver, kidneys, spleen, adrenal glands, sex organs, and brain).

### 2.3. Fat, Fatty Acid Profile, and Diterpenes in SCGs

Total fat content was quantified by Soxhlet extraction with petroleum ether following the procedure described by the AOAC Official Method 945.16. Results are expressed as % dry matter.

The fatty acid profile was obtained by gas chromatography (Agilent 7820A GC System equipped with Flame Ionization Detector, Waldbronn, Germany) analyses, calculated according to the ISO 12966-2:2017.

Diterpenes (cafestol and kahweol) were quantified using high liquid chromatography (Agilent 1200, Waldbronn, Germany) coupled to a quadruple triple mass spectrometer (Agilent G6410B, Waldbronn, Germany) (HPLC -QqQ). A Zorbax Eclipse XDB C18 993967-902 Agilent 150 mm × 5 μm × 4.6 mm column was used (Santa Clara, CA, USA). Two mobile phases of water and methanol in a 70% gradient were used with a flow of 1 mL/min. An atmospheric-pressure chemical ionization source (APCI) of positive polarity was used.

Samples were prepared by saponification of the dried fat with a mixture of 11.5 % KOH in ethanol water 55:45 *v*/*v* at 80 °C for 15 min. The unsaponifiable fraction was extracted with 1 mL of hexane. Aliquots of 0.5 mL of the solution were evaporated and resuspended in 1 mL of methanol (sample diluted 1:2). For quantification, pure cafestol and kahweol standards (Cayman Chemical Company, (Ann Arbor, MI, USA) were used. Each sample was prepared in duplicate and the standards were prepared following the same procedure carried out for the samples. Data were processed in the control software Masshunter Data Acquisition B.04.01 and Masshunter Qualitative Analysis B.07.00 (Waldbronn, Germany).

### 2.4. Bioaccesibility of Lipids and Diterpenes in SCGs

To study the effect of digestion on SCGs, an in vitro oral gastrointestinal digestion was carried out. SCGs were digested following the procedure described by Hollebeeck et al. (2013), with some modifications [18]. Salivary, gastric, and duodenal stages were performed in the same tube covered with aluminum foil. Approximately 1.2 g of SGCs were weighed. Conditions for each stage were: salivary stage (pH 6.9, 5 min, 3.9 U a-amylase/mL, aerobic), gastric stage (pH 2, 90 min, 71.2 U pepsin/mL, aerobic), and abiotic duodenal stage (pH 7, 150 min, 9.2 mg pancreatin and 55.2 mg bile extract/mL, aerobic). Obtained digests were centrifuged and separated in supernatant and precipitate. The content of fat and diterpenes was determined in the precipitate (non-digestible fraction, also called colonic fraction) and in the supernatant following the procedure described in Section 2.2. To simulate human intestinal reabsorption of bile salts, the soluble fraction was treated with cholestyramine resin (10% *w*/*v*) for 1 h at room temperature [19]. The resin was removed by centrifugation and gravimetric filtration.

### 2.5. Pilot Repeated Dose Animal Study

Male Wistar rats (average weight, 304 ± 15 g) were obtained from the Veterinary Unit at Universidad Rey Juan Carlos (URJC) (Alcorcón, Madrid, Spain) (*n* = 10). Animals were group-housed (2–4 rats/cage) in standard transparent cages (60 cm × 40 cm × 20 cm) under environmentally controlled conditions (20 °C and 60% humidity) with a 12-h light–dark cycle. Rats had free access to standard laboratory rat food (Harlan Laboratories Inc., Barcelona, Spain) and water. The study was approved by the Institutional Animal Ethics Committee (Reg. No. PROEX 059/18) of Community of Madrid, Spain. Rats were divided in two groups: controls (*n* = 6) and treated with SCGs (*n* = 4). Animals received SCGs (1 g/kg) or vehicle (Tween 80 in H_2_O, 1.8 mL/kg) by gavage once a day from Monday to Friday for 4 consecutive weeks. A fresh SCGs solution was prepared daily. Throughout the 4 weeks, general parameters were regularly evaluated (body weight, water and food intake, and appearance of animals). At the end of the study, feces were collected for the excretion study and animals were guillotined under anesthesia with sodium pentobarbital (2 mL/kg). Internal organs were obtained for histopathological examination for safety and metabolism evaluation.

#### 2.5.1. Metabolism Study

##### Determination of Fat and Cholesterol in Rat Livers

Livers from control and treated rats from the pilot animal study were collected for analysis. Liver fat was extracted by the method described by Aguilera et al. (2005) [20]. Frozen rat liver samples were thawed in refrigeration to a soft consistency. Two hundred milligrams of tissue were weighed in a 15 mL plastic test tube, 250 μL of distilled water was added, and the mixture was homogenized with an Ultra-Turrax (IKA, Staufen, Germany). Total fat was extracted with 1.5 mL of HCl (0.01 M), 0.1 mL of 1% MgCl_2_, and 5 mL of hexane:isopropanol (3:2). Samples were mixed for one minute and centrifuged at 5000 rpm for 10 min. The liquid phase was separated to another tube and centrifuged once more at 10,000 rpm for 10 min. Then, the supernatant obtained was separated and concentrated in a SpeedVac Vacuum Concentrator (Thermofisher, Asheville, NC, USA) to remove solvents for 12 h at low temperature and under vacuum. The total fat content of livers was calculated gravimetrically. Samples from each rat liver were prepared by duplicate.

For cholesterol determination, fat from the liver was dissolved in 1 mL of PBS with 50 μg of BHT/mL PBS, and then 2 mL of Triton X-110 were added. Cholesterol determination was performed spectrophotometrically using a commercial kit (Spinreact, Girona, Spain). Results are expressed in mg/dL.

##### Histopathological Examination

Samples from the gastrointestinal tract and liver were fixed in buffered formalin 10% (Panreac©, Barcelona, Spain, stabilized with methanol at pH 7) for 24 h at room temperature. Then, they were embedded in paraffin (Casa Alvarez, Madrid, Spain) using an automatic tissue processor (ASP300, Leica^®^, Wetzlar, Germany). Blocks were built in a block-forming unit (Leica© EG1140H, Wetzlar, Germany and Leica© EG1130, Wetzlar, Germany) and 4-micron thick sections were obtained (Leica© RM 2155, Wetzlar, Germany). Sections were deparaffinized in xylene, hydrated in alcohol and water, and stained with hematoxylin and eosin (Leica© SP4040, Wetzlar, Germany). Then, sections were dehydrated through an ascending series of alcohols, cleared in xylene, and finally, mounted with DPX (Nustain©, Nottingham, UK). Samples were studied qualitatively in 4 slices per animal under a Zeiss Axioskop 2 microscope equipped with the image analysis software package AxioVision 4.6 (Zeiss, Oberkochen, Germany).

#### 2.5.2. Lipid and Diterpenes Excretion

Number, weight, dry weight, fat, and diterpenes were determined in feces of control and treated rats collected at the end of the study following the methodology described in Section 2.3 of the present manuscript. 

#### 2.5.3. Dietary Fiber Effect Study (Gastrointestinal Motility)

The evaluation of the gastrointestinal motility was performed on the 1st, 14th, and 28th day of the study by radiographic, non-invasive, in vivo methods routinely used by the Pharmacology laboratory of the Universidad Rey Juan Carlos (URJC) [21,22]. Animals were fasted overnight and SCGs at 1 g/kg were given by gavage. Thirty minutes after SCGs administration, barium contrast (a mixture of 80% barium, 5% arabic gum, 1.6% sorbitol, 0.6% citric acid, and 0.8% citrate (*w*/*v*) in distilled water) was administered at 3 mL/rat, and radiographs (20 ms/shot, w/o anesthesia) were taken at 0, 1, 2, 3, 4, 6, 8, and 24 h (T0–T24) with a CS2100 (Carestream Dental, Madrid, Spain) digital X-ray apparatus (60 kV, 7 mA), recorded on Carestream Dental T-MAT G/RA film (15 × 30 cm) housed in a cassette provided with regular intensifying screen, and developed using an automatic processor (Kodak X-OMAT 2000, Rochester, NY, USA). For the analysis of the radiographs, after their digitalization, a semiquantitative scale was applied to the gastrointestinal region (stomach, small intestine, caecum, and colorectal region) of each rat, and at each time-point, scores between 0 and 12 were obtained and represented in the corresponding motility curves [21].

### 2.6. Statistical Analysis

Data are expressed as the mean ± standard deviation (SD) (in vitro studies) or standard error (SE) (in vivo studies). A one- or two-way analysis of variance (ANOVA) followed by Tukey’s or Bonferroni’s test (gastrointestinal motility analysis) for mean comparisons was used to highlight the significant differences among samples. A student’s t test was performed to compare means between two groups. Values of *p* < 0.05 (in vitro studies) and *p* < 0.1 (in vivo studies) were considered statistically significant. Statistical analyses were performed using SPSS Statistics 24 (IBM, Armonk, NY, USA) and GraphPad Prism program version 5.01 (GraphPad software, San Diego, CA, USA, for the gastrointestinal motility analysis).

## 3. Results and Discussion

### 3.1. Safety of SCGs

Coffee is susceptible to contamination by mycotoxins, toxic compounds that result from fungal secondary metabolism under certain conditions, which cause different toxicological effects in humans [23]. Studies carried out by Garcia-Moraleja et al. (2015) indicate that aflatoxin B1, enniantin B, and OTA are the most frequent mycotoxins present in coffee beverages [24]. Neither aflatoxin B1 nor enniantin B were detected, but OTA levels in SCGs were 2.31 ± 0.07 µg/kg. Commission Regulation (EC) No 123/2005 defined OTA limits as 5 μg/kg for roasted coffee and 10 μg/kg for soluble coffee [25]. Values of OTA obtained for instant Robusta SCGs used in this study were below the limit stablished by the European legislation.

Considering that OTA causes major health risks such as hepatotoxicity [26], an acute toxicity study in rats was carried out. A single oral administration of the SCGs at a dose of 2000 mg/kg b.w. resulted in no visible signs of toxicity, abnormal behavior, or mortality. Organ weights are shown in Table 1 and no significant differences (*p* > 0.05) were found among groups. Intake of an acute dose of SCGs (2000 mg/ kg b.w.) did not cause significant changes in histological parameters of vital organs (data not shown). To the best of our knowledge, mycotoxins and acute toxicity have been analyzed in this coffee byproduct for the first time.

### 3.2. In Vitro Bioaccesibility of Lipids and Diterpens Composing SCGs

Total fat content and the fatty acid profile of instant Robusta SCGs are shown in Table 2. The fat content of SCGs was 21.79 %, which is in accordance with that previously described by other authors for SCGs from soluble coffee [4,9]. Palmitic (62.58 mg/g), stearic (14.76 mg/g), oleic (20.38 mg/g), linoleic (87.36 mg/g), and arachidic (6.68 mg/g) acids were found in the SCGs sample, with 56 % of the total fatty acids being unsaturated. The presence of higher amounts of polyunsaturated fatty acids (PUFAs) than saturated fatty acids (SFAs) in oil is recognized as being more positive for human health [27]. In SCGs, PUFAs were only represented by linoleic acid. The PUFA/SFA ratio of SCGs was 1.038. Oils with PUFA/SFA ratios >1 have been reported to be less atherogenic and thrombogenic than those with ratios <1 due to the potential favorable reduction of serum cholesterol and atherosclerosis and prevention of heart diseases [3].

With regard to diterpene content, cafestol and kahweol levels in SCGs were 3.09 mg/g and 64.15 µg/g, respectively. Amounts of cafestol in the SCGs are in line with values obtained by Acevedo et al., (2013), but kahweol levels were lower (1.64 mg/g) [27]. However, kahweol values obtained in this study are similar to those present in Robusta coffee beans [28]. In addition, differences in values between studies are possible since levels of diterpenes in coffee beans depend on the species and storage conditions, as kahweol and cafestol are sensitive to acids, heat, and light [27].

After in vitro digestion, half of the total fat from the SCGs was bioaccessible, and half was excreted in the insoluble fraction (Table 2). It has been reported that SCGs possess a high oil-holding capacity, which is possibly due to the presence of lignin [6]. This capacity is a very desirable parameter for the functionality of a dietary fiber [6]. A total of 77% of unsaturated fatty acids remained bioaccessible after in vitro digestion, including linoleic (54.01 mg/g) and oleic acids (16.42 mg/g) as the most abundant, followed by the SFA palmitic acid (15.18 mg/g). With regard to diterpenes, low amounts of kahweol (7.09 µg/g) and cafestol (414.39 μg/g) were bioaccessible (Table 2). Knowledge about the absorption and metabolism of coffee diterpenes is limited. However, data obtained from animal studies treated with ^3^H-labeled cafestol have shown that cafestol is efficiently absorbed and partially metabolized by the gut, further metabolized by the liver, and then excreted into the bile [29]. It is known that cafestol is a cholesterol-raising compound in coffee beans that can directly regulate the expression of genes involved in cholesterol metabolism [12]. A very recent randomized crossover clinical trial has reported that unfiltered coffee consumption increased concentrations of serum lipids and total cholesterol levels in a healthy adult population, possibly due to cafestol [30]. According to these results, most of the unhealthy lipid compounds in instant SCGs may be bound to the dietary fiber and excreted in feces.

### 3.3. In Vivo Metabolism and Excretion of Lipids Composing SCGs

No adverse clinical signs (changes in body weight, food and water intake, hydration, and animal behavior) or mortality were observed in animals treated with SCGs (1 g/kg b.w.) for 28 days. Altogether, the results obtained in the study seem to indicate that the administration of SCGs at 1 g/kg b.w. for 4 weeks was not noxious to the animals. During the pilot study, changes in body weight and food and water intakes were comparable in both groups throughout the experiment (*p* > 0.05). The mean weights of control and treated animals at the beginning of the study were 312 ± 17 g and 298 ± 9 g, respectively. Throughout the four weeks of the pilot study, there was an increase in weight in both groups, without showing significant differences in this parameter (*p* > 0.05) due to the intake of SCGs. The food and water intake of the control group was 23 ± 4 g/day/rat and 34 ± 9 mL/day/rat, and that of the treated group was 20 ± 3 g/day/rat and 30 ± 8 mL/day/rat, respectively. No statistically significant differences were observed between groups (*p* > 0.05).

At the end of the study, vital organs were weighed, and no significant changes were observed between control and treated rats (*p* > 0.05). In addition, organs were subjected to histopathological examination, and no significant damage was observed in their architecture.

**Figure 1:**
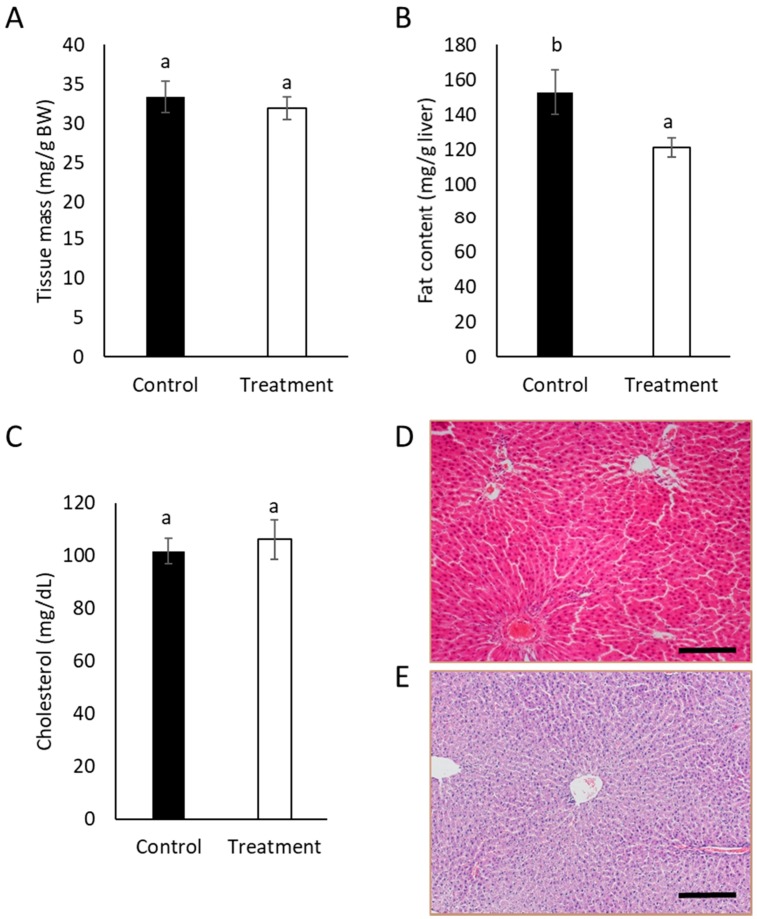
(**A**) Tissue mass (g/g BW), (**B**) fat content (mg/g liver), and (**C**) cholesterol content (mg/dL) of livers from control and treated animals. Data expressed as mean ± SE. Different letters indicate significant differences between groups (t test, *p* < 0.1). Hematoxilin-eosin staining of formalin-fixed samples of the liver of a control rat (**D**) and a rat treated with SCGs (**E**). Samples were obtained on the 29th day. Bar 200 μm.

The liver is a major organ for the synthesis, metabolism, storage, and distribution of lipids, and it plays an essential role in regulating energy metabolism [31]. Thus, liver from treated and control rats was analyzed after the pilot study. Treatment of animals with instant SCGs for 28 days did not cause significant differences (*p* > 0.1) in liver weight (Figure 1A). In contrast, fat content in treated rats was significantly lower than that of control rats (*p* < 0.1), although no lipid droplets or histological damage was observed in the liver (Figure 1D), and cholesterol levels between control rats and those treated with SCGs did not differ significantly (*p* > 0.1) (Figure 1C).

Nonalcoholic fatty liver disease is related to obesity and metabolic syndrome and is characterized by excess of fat deposition in the liver, which leads to an increased risk of type 2 diabetes mellitus, hyperlipidemia, and insulin resistance [31]. No previous studies have reported the effect of SCGs on liver health, but there is increasing evidence regarding the protective effects of coffee consumption in the development of liver disease due to hepatitis B and C, nonalcoholic fatty liver disease, and alcoholic liver disease [32]. The main responsible compounds proposed for the hepatoprotective effects of coffee are caffeine, phenolic compounds, and melanoidins. These compounds are responsible for antioxidant effects at the hepatic level that prevent free radical tissue damage by reducing reactive oxygen species, which play a central part in the inflammation processes characterizing nonalcoholic fatty liver disease and other liver diseases [33]. Since these compounds are also present in SCGs [4], this byproduct may be a good candidate for the treatment of metabolic syndrome. Further studies, using specific models, are needed to confirm its capacity to prevent hepatic steatosis.

**Figure 2:**
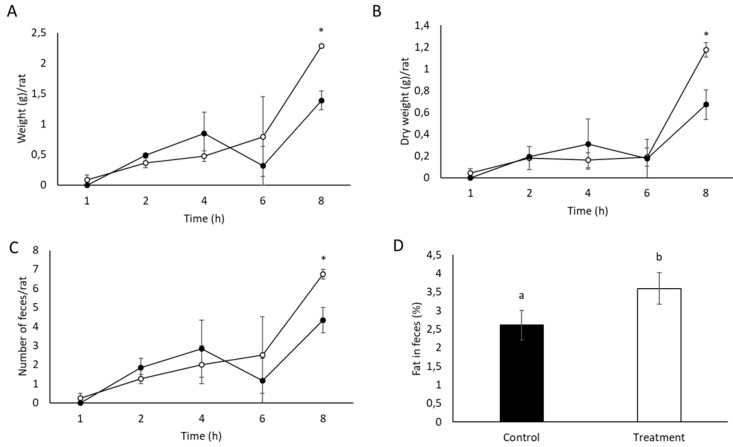
Weight (**A**), dry weight (**B**), number (**C**), and fat content (**D**) in feces collected the 28th day of the study during the last X-ray session. Data represent the mean ± SE. Asterisks * indicate significant differences between groups at each hour (t test, *p* < 0.1). In the analysis of fat content of feces, different letters denote significant differences (t test, *p* < 0.1).

Figure 2 shows the weight, number, and fat content of feces collected the 28th day of the study from control and treated rats. Wet and dry weight and number of feces were significantly increased (*p* < 0.1) in rats treated with SCGs when compared to the control group. In addition, feces of rats treated with SCGs showed a significantly higher fat content (*p* < 0.1) than feces of the control group, suggesting higher excretion of fat. These results are in accordance with those obtained in the in vitro oral gastrointestinal digestion assays (Table 2), which also showed a tendency of higher fat excretion. In contrast, no cafestol or kahweol were detected in feces.

### 3.4. Gastrointestinal Motility

**Figure 3:**
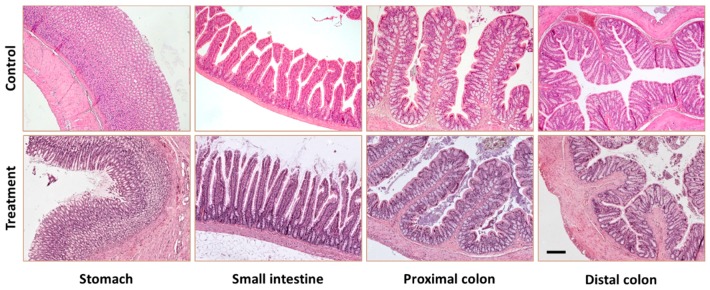
Histological staining showing stomach, small intestine, proximal and distal colon architecture (hematoxilin-eosin staining of formalin-fixed samples) from control rats and rats treated with SCGs. Samples were obtained on the 29th day. Bar 200 μm.

Tissue samples of organs from the gastrointestinal tract of treated rats are shown in Figure 3. As can be observed, the different gastrointestinal regions showed normal morphology, suggesting no significant damage caused by the repeated intake of SCGs (1 g/kg b.w.) during 28 days.

**Figure 4:**
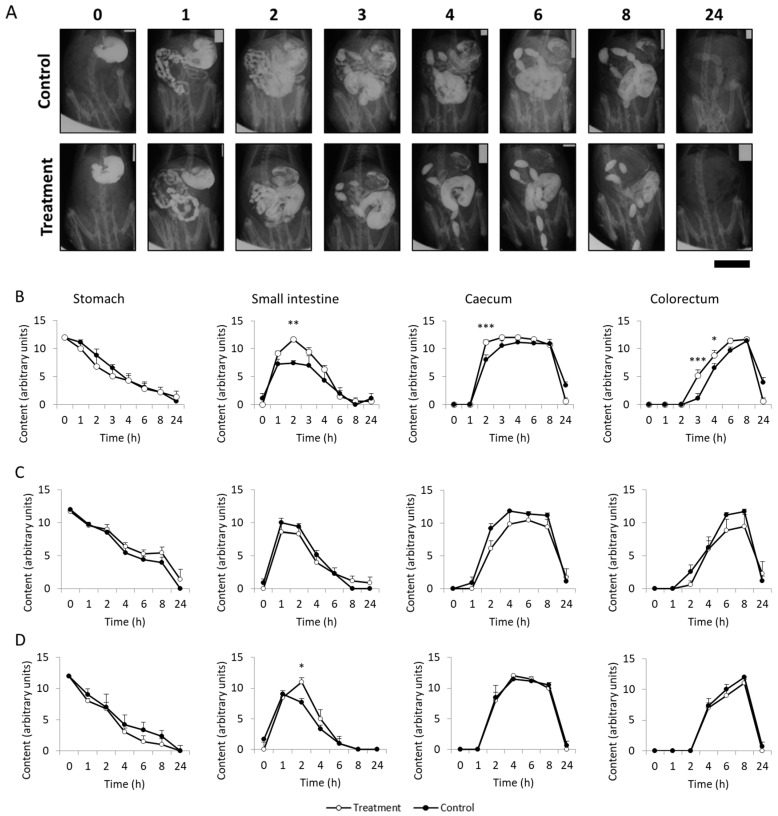
(**A**) Radiological images of X-rays from the 1st day of treatment of controls and rats treated with SCGs, taken immediately and 1, 2, 3, 4, 6, 8, and 24 h after barium administration. Scale bar = 3 cm. Radiological analysis of X-rays taken on the 1st (**B**), 14th (**C**), and 28th (**D**) day of treatment according to Cabezos et al. (2008) [21]. Data represent the mean ± SE. * (*p* < 0.05), ** (*p* < 0.005), *** (*p* < 0.001) (two-way ANOVA followed by Bonferroni test).

The fiber effect of SCGs was studied using radiographic analyses performed on the 1st, 14th, and 28th day of the study. Results of the radiographic study carried out on the 1st day of the study are shown in Figure 4. Control rats showed normal motor function in all gastrointestinal regions and radiographic sessions. SCGs did not significantly alter the normal progressive gastric emptying in any of the X-ray sessions compared to the control group (*p* > 0.05). In contrast, SCGs significantly accelerated (*p* < 0.05) intestinal transit during the first X-ray session. Results of the semiquantitative analysis of the small intestine showed a filling phase (0–1 h), a plateau (1–3 h), and a progressive emptying phase (3–8 h) in the control group (Figure 4B). However, animals treated with SCGs showed a filling maximum at 2 h that was significantly higher than that of control rats (*p* < 0.005). With regard to the caecum, significant differences were observed (*p* < 0.001), since it took 4 h for the caecum of the control animals to be completely filled, whereas that of the treated animals was already filled in 2 h (Figure 4B).

Considering the colorectum curve, the control group began to form fecal pellets 4 h after barium administration and reached the maximum score at the 8 hour of the radiographic session. However, SCGs significantly accelerated fecal pellet formation at 3 and 4 h after contrast administration (*p* < 0.001 and *p* < 0.05, respectively).

In contrast, on X-rays taken on the 14th and 28th day of treatment, intestinal motor function overlapped with that of controls (Figure 4C,D). This suggests that upon chronic treatment, tolerance may develop to the intestinal stimulating effect of SCGs [34]. Importantly, no sign of impaired motility was found in any animal. To the best of our knowledge, no previous studies have reported the effect of SCGs on intestinal motility.

Data hereby reported support the dietary fiber effect of SCGs and their potential for modulating lipid metabolism, which may be associated to their fat-holding capacity. Other gastrointestinal health-promoting properties have been attributed to SCGs. Lopez-Barrera et al. (2016) reported that SCGs can be fermented by colon microbiota producing short chain fatty acids (SCFAs) with anti-inflammatory properties [35]. SCFAs generated after fermentation of SCGs had the ability to suppress NO production and inhibited inflammatory mediators (cytokines IL-10, CCL-17, CXCL9, IL-1b, and IL-5) in murine macrophage cells [35,36]. The in vivo effects of SCFAs on colonic motility have been controversial. However, it has been shown that luminal administration of SCFAs (10–200 mM) stimulates colonic motility [37]. Lopez-Barrera et al. (2016) reported the generation of SCFAs in concentrations higher than 10 mM during colonic fermentation of SCGs from medium and dark roasted coffee beans [35]. Results obtained in the present research regarding gastrointestinal motility might be influenced by SCFAs released during SCGs fermentation.

## 4. Conclusions

Acute and repeated treatments of Wistar rats with SCGs were not noxious to the animals. Most of the SFAs and diterpenes in instant SCGs were not bioaccesible after in vitro digestion. Results seem to indicate that they may be bound to the SCGs dietary fiber and excreted in feces, reducing fat accumulation in liver. These results suggest that SCGs might be used as a sustainable, safe, and healthy food ingredient for preventing hepatic steatosis and treating metabolic syndrome. Further studies in humans should be conducted to confirm this hypothesis.

## Figures and Tables

**Table 1:** Organ weights in female rats included in the control group and in the group exposed to the limit dose of 2000 mg/kg b.w.

Organs	Control	Treatment
Heart	1.02 ± 0.23 ^a^	0.90 ± 0.07 ^a^
Lungs	1.90 ± 0.25 ^a^	1.56 ± 0.23 ^a^
Liver	9.72 ± 1.21 ^a^	9.94 ± 1.25 ^a^
Kidneys	2.08 ± 0.11 ^a^	2.00 ± 0.19 ^a^
Spleen	0.62 ± 0.15 ^a^	0.60 ± 0.10 ^a^
Thymus	0.66 ± 0.15 ^a^	0.58 ± 0.13 ^a^
Adrenal glands	0.16 ± 0.09 ^a^	0.18 ±0.08 ^a^
Uterus	1.52 ± 0.67 ^a^	1.36 ± 0.18 ^a^
Brain	1.80 ± 0.18 ^a^	1.70 ± 0.20 ^a^

Values represent mean ± SD (*n* = 5). ^a^ Different letters indicate significant differences (t test, *p* < 0.05).

**Table 2:** Total fat (%), fatty acid profile (mg/g sample and % of total), and diterpenes (μg/g sample) of spent coffee grounds (SCGs) and their bioaccessible and excreted fraction obtained after in vitro oral-gastrointestinal digestion.

Analysis	SCGs		Bioaccessible Fraction		Fiber Fraction	
**Total Fat (%)**	21.79 ± 0.23 ^b^		11.87 ± 1.66 ^a^		14.21 ± 0.82 ^a^	
**Fatty Acid**	**(mg/g)**	**(%)**	**(mg/g)**	**(%)**	**(mg/g)**	**(%)**
C14:0	0.21 ± 0.01 ^c^	0.11	0.12 ± 0.01 ^a^	0.13	0.19 ± 0.01 ^b^	0.17
C15:0	0.09 ± 0.00 ^b^	0.05	0.06 ± 0.00 ^a^	0.06	0.11 ± 0.01 ^c^	0.10
C16:0	62.58 ± 2.38 ^c^	31.58	15.18 ± 0.94 ^a^	15.59	41.56 ± 0.74 ^b^	37.87
C16:1n7	0.14 ± 0.01 ^a^	0.07	0.39 ± 0.03 ^c^	0.40	0.21 ± 0.01 ^b^	0.19
C17:0	0.17 ± 0.01 ^b^	0.09	0.11 ± 0.01 ^a^	0.11	0.28 ± 0.01 ^c^	0.26
C18:0	14.76 ± 0.56 ^b^	7.45	3.67 ± 0.30 ^a^	3.77	11.67 ± 0.16 ^c^	10.64
C18:1n7c	0.77 ± 0.03 ^b^	0.39	0.88 ± 0.06 ^c^	0.90	0.54 ± 0.03 ^a^	0.50
C18:1n9c	20.38 ± 0.84 ^c^	10.28	16.42 ± 1.23 ^b^	16.84	10.62 ± 0.43 ^a^	9.67
C18:2n6c	87.36 ± 3.73 ^c^	44.07	54.01 ± 4.82 ^b^	55.34	36.85 ± 0.99 ^a^	33.57
C18:3n3	1.75 ± 0.09 ^c^	0.88	1.03 ± 0.09 ^b^	1.05	0.76 ± 0.03 ^a^	0.69
C18:3n6	n.d.	0.00	0.08 ± 0.01 ^b^	0.08	0.04 ± 0.01 ^a^	0.04
C20:0	6.68 ± 0.26 ^c^	3.37	1.23 ± 0.10 ^a^	1.26	3.90 ± 0.07 ^b^	3.55
C20:1n9	0.83 ± 0.04 ^c^	0.42	0.44 ± 0.03 ^b^	0.45	0.32 ± 0.01 ^a^	0.30
C20:2n6	0.12 ± 0.01 ^b^	0.06	0.17 ± 0.01 ^c^	0.17	0.09 ± 0.01 ^a^	0.08
C20:3n6	0.21 ± 0.01 ^c^	0.10	0.04 ± 0.00 ^a^	0.04	0.13 ± 0.00 ^b^	0.12
C20:4n6	n.d.	0.00	2.59 ± 0.37 ^b^	2.65	0.99 ± 0.10 ^a^	0.90
C20:5n3	0.14 ± 0.01 ^c^	0.07	0.11 ± 0.01 ^b^	0.11	0.06 ± 0.01 ^a^	0.05
C21:0	n.d.	0.00	0.10 ± 0.01 ^b^	0.10	0.05 ± 0.01 ^a^	0.04
C22:0	0.96 ± 0.03 ^c^	0.49	0.20 ± 0.02 ^a^	0.21	0.59 ± 0.01 ^b^	0.54
C22:1n9	0.21 ± 0.01 ^b^	0.11	0.10 ± 0.01 ^a^	0.11	0.10 ± 0.01 ^a^	0.09
C22:4n6	n.d.	0.00	0.11 ± 0.02 ^b^	0.11	0.05 ± 0.00 ^a^	0.04
C22:5n3	n.d.	0.00	0.20 ± 0.03 ^b^	0.21	0.10 ± 0.01 ^a^	0.09
C22:6n3	n.d.	0.00	0.10 ± 0.01 ^b^	0.10	0.04 ± 0.01 ^a^	0.04
C23:0	0.25 ± 0.00 ^c^	0.13	0.06 ± 0.01 ^a^	0.07	0.17 ± 0.01 ^b^	0.15
C24:0	0.58 ± 0.02 ^c^	0.29	0.13 ± 0.02 ^a^	0.13	0.35 ± 0.00 ^b^	0.32
SFA (%)	43.54 ± 0.11 ^c^	-	21.43 ± 0.75 ^a^		53.64 ± 0.49 ^b^	
MUFA (%)	11.27 ± 0.01 ^b^	-	18.70 ± 0.22 ^c^		10.74 ± 0.21 ^a^	
PUFA (%)	45.19 ± 0.11 ^b^	-	59.87 ± 0.97 ^c^		35.62 ± 0.29 ^a^	
**Diterpenes (µg/g)**						
Cafestol	3095.39 ± 518.81 ^b^		414.39 ± 25.80 ^a^		1029.90 ± 55.51 ^a^	
Kahweol	64.19 ± 9.35 ^b^		7.09 ± 1.68 ^a^		22.50 ± 0.34 ^a^	

n.d., non-detected; SFA, saturated fatty acids; MUFA, monounsaturated fatty acids; PUFA, polyunsaturated fatty acids. Results are expressed as mean ± SD. Different letters indicate significant differences (Tukey test, *p* < 0.05).

## References

[1] Padmapriya R., Tharian J., Thirunalasundari T. (2013). Coffee waste management-An overview. Int. J. Curr. Sci..

[2] Murthy P.S., Madhava Naidu M. (2012). Sustainable management of coffee industry by-products and value addition—A review. Resour. Conserv. Recycl..

[3] Campos-Vega R., Loarca-Piña G., Vergara-Castañeda H., Oomah B.D. (2015). Spent coffee grounds: A review on current research and future prospects. Trends Food Sci. Technol..

[4] Martinez-Saez N., Tamargo García A., Domínguez Pérez I., Rebollo-Hernanz M., Mesías M., Morales F.J., Martín-Cabrejas M.A., del Castillo M.D. (2017). Use of spent coffee grounds as food ingredient in bakery products. Food Chem..

[5] del Castillo M.D., Martinez-Saez N., Ullate M. (2014). Healthy Bakery Products with High Level of Dietary Antioxidant Fibre. International Patent.

[6] Ballesteros L.F., Teixeira J.A., Mussatto S.I. (2014). Chemical, Functional, and Structural Properties of Spent Coffee Grounds and Coffee Silverskin. Food Bioprocess Technol..

[7] Mussatto S.I., Carneiro L.M., Silva J.P.A., Roberto I.C., Teixeira J.A. (2011). A study on chemical constituents and sugars extraction from spent coffee grounds. Carbohydr. Polym..

[8] Simões J., Nunes F.M., Domingues M.R., Coimbra M.A. (2013). Extractability and structure of spent coffee ground polysaccharides by roasting pre-treatments. Carbohydr. Polym..

[9] Pujol D., Liu C., Gominho J., Olivella M.À., Fiol N., Villaescusa I., Pereira H. (2013). The chemical composition of exhausted coffee waste. Ind. Crops Prod..

[10] Calligaris S., Munari M., Arrighetti G., Barba L. (2009). Insights into the physicochemical properties of coffee oil. Eur. J. Lipid Sci. Technol..

[11] George S.E., Ramalakshmi K., Rao L.J.M. (2008). A perception on health benefits of coffee. Crit. Rev. Food Sci. Nutr..

[12] Ricketts M.-L., Boekschoten M.V., Kreeft A.J., Hooiveld G.J.E.J., Moen C.J.A., Müller M., Frants R.R., Kasanmoentalib S., Post S.M., Princen H.M.G. (2007). The Cholesterol-Raising Factor from Coffee Beans, Cafestol, as an Agonist Ligand for the Farnesoid and Pregnane X Receptors. Mol. Endocrinol..

[13] Silva V.M., Vieira G.S., Hubinger M.D. (2014). Influence of different combinations of wall materials and homogenisation pressure on the microencapsulation of green coffee oil by spray drying. Food Res. Int..

[14] del Castillo M.D., Fernandez-Gomez B., Martinez-Saez N., Iriondo-DeHond A., Mesa M.D., Farah A. (2019). Coffee By-Products. Coffee: Chemistry, Quality and Health Implications.

[15] Kovalcik A., Obruca S., Marova I. (2018). Valorization of spent coffee grounds: A review. Food Bioprod. Process..

[16] Vázquez-Sánchez K., Martinez-Saez N., Rebollo-Hernanz M., del Castillo M.D., Gaytán-Martínez M., Campos-Vega R. (2018). In Vitro health promoting properties of antioxidant dietary fiber extracted from spent coffee (Coffee arabica L.) grounds. Food Chem..

[17] Iriondo-DeHond A., Aparicio García N., Velazquez Escobar F., San Andres M.I., Sanchez-Fortun S., Blanch G.P., Fernandez-Gomez B., Guisantes Batan E., del Castillo M.D. (2019). Validation of coffee by-products as novel food ingredients. Innov. Food Sci. Emerg. Technol..

[18] Hollebeeck S., Borlon F., Schneider Y.-J., Larondelle Y., Rogez H. (2013). Development of a standardised human in vitro digestion protocol based on macronutrient digestion using response surface methodology. Food Chem..

[19] Edwards A.D., Slater N.K.H. (2009). Protection of live bacteria from bile acid toxicity using bile acid adsorbing resins. Vaccine.

[20] Aguilera C.M., Ramirez-Tortosa C.L., Quiles J.L., Yago M.D., Martínez-Burgos M.A., Martínez-Victoria E., Gil Á., Ramirez-Tortosa M.C. (2005). Monounsaturated and ω-3 but not ω-6 polyunsaturated fatty acids improve hepatic fibrosis in hypercholesterolemic rabbits. Nutrition.

[21] Cabezos P.A., Vera G., Castillo M., Fernández-Pujol R., Martín M.I., Abalo R. (2008). Radiological study of gastrointestinal motor activity after acute cisplatin in the rat. Temporal relationship with pica. Auton. Neurosci..

[22] Abalo R., Cabezos P.A., López-Miranda V., Vera G., González C., Castillo M., Fernández-Pujol R., Martín M.I. (2009). Selective lack of tolerance to delayed gastric emptying after daily administration of WIN 55,212-2 in the rat. Neurogastroenterol. Motil..

[23] Afsah-Hejri L., Jinap S., Hajeb P., Radu S., Shakibazadeh S. (2013). A Review on Mycotoxins in Food and Feed: Malaysia Case Study. Compr. Rev. Food Sci. Food Saf..

[24] Garcia-Moraleja A., Font G., Manes J., Ferrer E. (2015). Analysis of mycotoxins in coffee and risk assessment in Spanish adolescents and adults. Food Chem. Toxicol..

[25] European Commission (2005). Commission Regulation (EC) No 123/2005 of 26 January 2005 amending Regulation (EC) No 466/2001 as regards ochratoxin A. Off. J. Eur. Union.

[26] Kőszegi T., Poór M. (2016). Ochratoxin A: Molecular interactions, mechanisms of toxicity and prevention at the molecular level. Toxins.

[27] Acevedo F., Rubilar M., Scheuermann E., Cancino B., Uquiche E., Garcés M., Inostroza K., Shene C. (2013). Spent coffee grounds as a renewable source of bioactive compounds. J. Biobased Mater. Bioenergy.

[28] De Roos B., Meyboom S., Kosmeijer-Schuil T.G., Katan M.B. (1998). Absorption and urinary excretion of the coffee diterpenes cafestol and kahweol in healthy ileostomy volunteers. J. Intern. Med..

[29] Van Cruchten S.T.J., De Waart D.R., Kunne C., Hooiveld G.J.E.J., Boekschoten M.V., Katan M.B., Oude Elferink R.P.J., Witkamp R.F. (2010). Absorption, distribution, and biliary excretion of cafestol, a potent cholesterol-elevating compound in unfiltered coffees, in mice. Drug Metab. Dispos..

[30] Eren F.H., Besler H.T. (2019). A 4-week consumption of light or dark roast unfiltered (Turkish) coffee affects cardiovascular risk parameters of homocysteine and cholesterol concentrations in healthy subjects: A randomized crossover clinical trial. Prog. Nutr..

[31] Tirosh O. (2018). Hypoxic Signaling and Cholesterol Lipotoxicity in Fatty Liver Disease Progression. Oxid. Med. Cell. Longev..

[32] Nieber K. (2017). The Impact of Coffee on Health Author Pharmacokinetics and Mode of Action Bioactive Components in Coffee. Planta Med..

[33] Grosso G., Godos J., Galvano F., Giovannucci E.L. (2017). Coffee, Caffeine, and Health Outcomes: An Umbrella Review. Annu. Rev. Nutr..

[34] Muller-Lissner S.A., Kamm M.A., Scarpignato C., Wald A. (2005). Myths and Misconceptions About Chronic Constipation. Am. J. Gastroenterol..

[35] López-Barrera D.M., Vázquez-Sánchez K., Loarca-Piña M.G.F., Campos-Vega R. (2016). Spent coffee grounds, an innovative source of colonic fermentable compounds, inhibit inflammatory mediators in vitro. Food Chem..

[36] Campos-Vega R., Vázquez-Sánchez K., López-Barrera D., Loarca-Piña G., Mendoza-Díaz S., Oomah B.D. (2015). Simulated gastrointestinal digestion and in vitro colonic fermentation of spent coffee (Coffea arabica L.): Bioaccessibility and intestinal permeability. Food Res. Int..

[37] Fukumoto S., Tatewaki M., Yamada T., Fujimiya M., Mantyh C., Voss M., Eubanks S., Harris M., Pappas T.N., Takahashi T. (2003). Short-chain fatty acids stimulate colonic transit via intraluminal 5-HT release in rats. Am. J. Physiol. Integr. Comp. Physiol..

